# Central serous chorioretinopathy with angioid streaks: a rare combination

**DOI:** 10.3205/oc000083

**Published:** 2018-02-22

**Authors:** Ambreen Sarmad, Fadi Alfaqawi, Monali Chakrabarti, Samer Elsherbiny

**Affiliations:** 1 Birmingham and Midland Eye Centre, Birmingham, Great Britain

**Keywords:** central serous chorioretinopathy, angioid streaks, choroidal neo-vascularizartion, CNV, photodynamic therapy

## Abstract

**Purpose:** This rare case shows the presence of both angioid streaks (AS) and central serous chorioretinopathy (CSC) in the same eye.

**Methods:** A 41-year-old Caucasian male who also has a positive family history of AS was diagnosed with angioid streaks. He was followed for few years, later developed CSC in his good eye.

**Results:** Fundus fluorescein led to the diagnosis of CSC and indocyanine green angiography ruled out the possibility of idiopathic polypoidal choroidal vasculopathy (IPCV). The CSC followed a chronic course of non-resolution and finally half fluence photodynamic therapy was performed. Unfortunately, there was still some deterioration of vision with poor response.

**Conclusion:** There is no known correlation between the two disorders and their presence in one eye has not been reported to our knowledge.

## Introduction

Angioid streaks (AS) are a rare disorder characterized by grey or dark red linear lesions with irregular serrated edges beneath the normal retinal that intercommunicate in an intertwining irregular fashion around the optic disc, radiating outwards. These are caused by breaks or dehiscence in a thickened, calcified, and abnormally brittle Bruch’s membrane [1]. Complications include choroidal neo-vascularizartion (CNV), sub-retinal haemorrhage and visual loss, causing metamorphosia due to macular involvement.

Central serous chorioretinopathy (CSC) is a predominantly self-limiting disorder of the outer blood-retinal barrier secondary to a focal retinal pigment epithelium (RPE) dysfunction, more commonly occurring in middle-aged men. 

We present a case of AS associated with CSC in the same eye. To our knowledge no correlation between the two conditions has been described in the literature previously. 

## Case description

A Caucasian male was diagnosed as a case of AS in 2006 at our department, at the age of 41. He had a positive family history of the disease, with his brother diagnosed before him with the same disease. At the time of initial presentation, his right eye’s best-corrected VA was 3/60 with a disciform scar secondary to neovascular membrane and the left eye had a VA of 6/9. His right eye vision was considered to be too low for any kind of treatment and he was discharged with advice on self-monitoring with an Amsler Grid. 

Over the next few years he kept on visiting the department intermittently for new symptoms picked up on self-monitoring. However, his vision in the left eye remained stable, at 6/9. During this time, he also developed a retinal tear on the left eye due to posterior vitreous detachment, for which he had successful laser retinopexy. 

**Figure 1:**
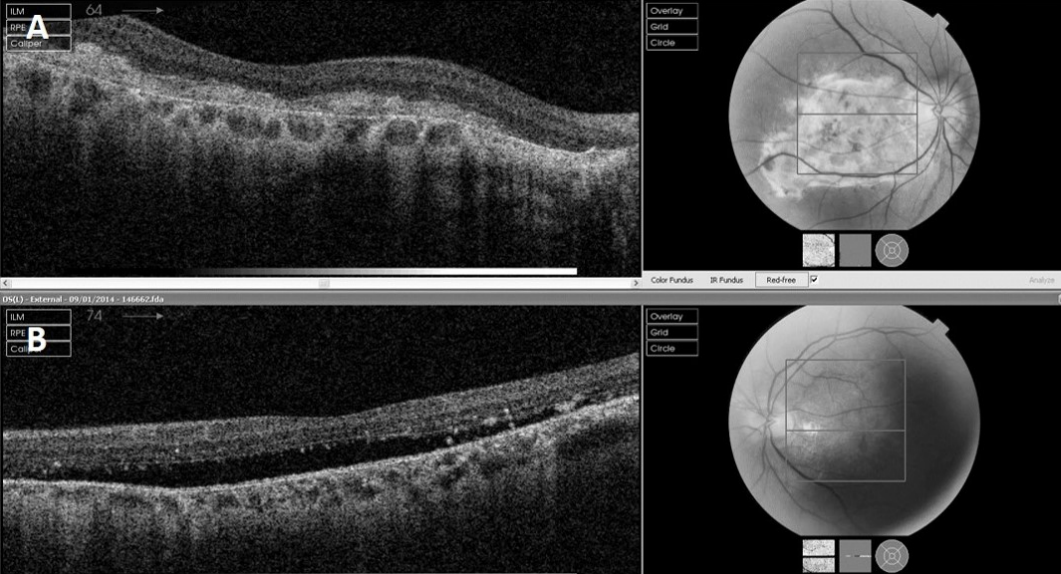
A) Right eye showing disciform scar at initial presentation. B) Left eye showing neurosensory elevation at initial presentation.

In 2011, he presented with a mild central blur on the grid. At this time, a significant neurosensory elevation of the macula was picked up on fundoscopy and optical coherence tomography (OCT) (Figure 1 (Fig. 1)). This was diagnosed as CSC on the basis of fundus fluorescein angiography (FFA), which did not reveal any CNV with vision remaining around 6/9, fluctuating but not progressively deteriorating. Indocyanine green angiography (ICG) was done to rule out any idiopathic polypoidal choroidal vasculopathy (IPCV), which was not present.

**Figure 2:**
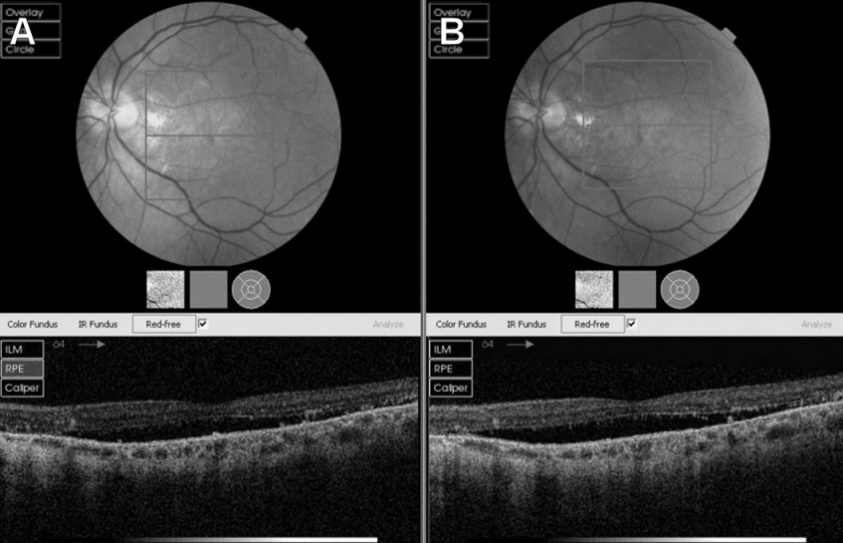
A) Left eye showing poor response post-PDT at 4 months. B) Left eye showing persistent neurosensory elevation post-PDT at 6 months.

After being followed up for almost 3 years with non-resolving CSC, his left eye VA dropped permanently to 6/12 and he started to struggle with his work due to poor vision. At that point, half fluence photodynamic therapy with vereteporfin (PDT) was carried out. Unfortunately, the PDT showed poor response as well and his vision still showed mild deterioration overtime (Figure 2 (Fig. 2)).

## Discussion

Although both AS and CSC have been well described, to our knowledge they have not previously been reported simultaneously in the same eye of a single patient with angiographic exclusion of a CNV. We therefore believe that this is the first report of this co-pathology.

A long list of diseases in addition to Ehlers-Danlos syndrome and Pseudoxanthoma elasticum have been associated with AS. However, very few of them have been associated with CSC [2]. Various other factors including “type A” personality (high levels of stress), pregnancy, Cushing’s disease, antibiotics, hypertension, alcohol, organ transplant, and steroids have been implicated to induce or aggravate CSC [3]. 

AS, on its own, does not require any treatment. However, its complications can be sight-threatening, hence requiring intervention. This can occur with macular involvement or choroidal neovascularization. Despite choroidal neovascularization developing in a large number of patients, treatment with laser photocaggulation, PDT and macular translocation do not show long-term visual benefit [2]. Intravitreal vascular endothelial growth factor inhibitors (anti-VEGF) are, currently considered by many, the most effective treatment of CNV due to AS [4].

CSC tends to occur asymmetrically, bilaterally and has a self-limiting course in the vast majority of patients. Various treatment options suggested include reduced fluence PDT [5], anti-VEGF therapy, micro-pulsed diode laser, and the cessation of steroid medications. 

Our choice of PDT for the left eye was based on three factors. The first was subjective and objective reduction in vision. The second was the exclusion of CNV, hence ruling out anti-VEGF therapy. The third is the presence of published evidence to suggest that PDT may be a useful treatment option for chronic CSC at least in the short-term [6]. Transient loss of the photoreceptor outer segments has been reported as a cause of severe visual loss after PDT for CSC [7] but in this patient a progressive deterioration of vision may be related to AS and CSC itself rather than the effect of PDT. Hansen at al. suggested that the cause of visual loss in AS may principally be related to underlying retinal detachment and persistent serous detachment [8]. Despite visual deterioration, we are not considering any anti-VEGF therapy at present as there is no evidence of neo-vascularization. 

## Notes

### Competing interests

The authors declare that they have no competing interests.
